# Influence of Single-Nucleotide Polymorphisms on Clinical Outcomes of Capecitabine-Based Chemotherapy in Colorectal Cancer Patients: A Systematic Review

**DOI:** 10.3390/cancers15061821

**Published:** 2023-03-17

**Authors:** Yasmin Cura, Cristina Pérez-Ramírez, Almudena Sánchez-Martín, Cristina Membrive-Jimenez, María Isabel Valverde-Merino, Encarnación González-Flores, Alberto Jiménez Morales

**Affiliations:** 1 Pharmacy Service, Pharmacogenetics Unit, Hospital Universitario Virgen de las Nieves, Avda. de las Fuerzas Armadas 2, 18004 Granada, Spain; 2 Department of Biochemistry and Molecular Biology II, José Mataix Institute of Nutrition and Food Technology, Center for Biomedical Research, Universidad de Granada, Avda. del Conocimiento s/n, 18016 Granada, Spain; 3 Pharmaceutical Care Research Group, Facultad de Farmacia, Universidad de Granada, Campus de la Cartuja, 18071 Granada, Spain; 4 Medical Oncology, Hospital Universitario Virgen de las Nieves, Avda. de las Fuerzas Armadas 2, 18004 Granada, Spain; 5 Biosanitary Research Institute of Granada, Ibs.Granada, Avda. de Madrid, 15, 18012 Granada, Spain

**Keywords:** colorectal cancer, capecitabine, clinical outcomes, pharmacogenetics, single-nucleotide polymorphisms

## Abstract

**Simple Summary:**

Colorectal cancer is one of the most prevalent neoplasms worldwide. Capecitabine is an oral fluoropyrimidine widely used to treat colorectal cancer in early and advanced stages. However, it shows high interindividual variability in its effectiveness and safety. This variability may be due to genetic variants in proteins involved in the pharmacokinetics and pharmacodynamics of the drug. Currently, only four variants of the *DPYD* gene are clinically relevant for the prediction of severe toxicity, and there are no validated predictive biomarkers of capecitabine effectiveness. Therefore, the search of potential predictive genetic biomarkers to personalize and optimize capecitabine therapy remains necessary. The aim of this study was to systematically review the literature published in the last 10 years on the influence of single-nucleotide polymorphisms in the main genes involved in capecitabine pharmacokinetics and pharmacodynamics on therapy outcomes in patients with colorectal cancer.

**Abstract:**

The aim of this systematic review was to provide a comprehensive overview of the literature published in the last decade on the association of single-nucleotide polymorphisms in genes involved in the pharmacodynamic and pharmacokinetic pathways of capecitabine with treatment outcomes among colorectal cancer patients. A systematic search of the literature published in the last 10 years was carried out in two databases (Medline and Scopus) using keywords related to the objective. Quality assessment of the studies included was performed using an assessment tool derived from the Strengthening the Reporting of Genetic Association (STREGA) statement. Thirteen studies were included in this systematic review. Genes involved in bioactivation, metabolism, transport, mechanism of action of capecitabine, DNA repair, and folate cycle were associated with toxicity. Meanwhile, genes related to DNA repair were associated with therapy effectiveness. This systematic review reveals that several SNPs other than the four *DPYD* variants that are screened in clinical practice could have an impact on treatment outcomes. These findings suggest the identification of future predictive biomarkers of effectiveness and toxicity in colorectal cancer patients treated with capecitabine. However, the evidence is sparse and requires further validation.

## 1. Introduction

Colorectal cancer (CRC) is one of the most prevalent malignancies worldwide. In the United States, it is estimated that the incidence of CRC in 2022 will be 151,030 new cases [1]. According to data provided by the International Agency for Research on Cancer (IARC), CRC is the second leading cause of cancer death worldwide, accounting for 9.4% of deaths [2].

Capecitabine, an oral fluoropyrimidine (FP), is a prodrug of 5-fluorouracil (5-FU) used to treat CRC in both early and advanced stages [3]. It can be used as monotherapy and in combination with other chemotherapeutic agents, biologics, or radiotherapy (RT) [4,5]. Despite its widespread use, capecitabine therapy shows high interindividual variability in its effectiveness and safety. The observed variability may be due to patients’ clinical or demographic factors, including genetic factors [6]. Genetic variants that affect the activity of proteins involved in the pharmacokinetics (PK) and pharmacodynamics (PD) of capecitabine could cause different intensities and durations of the observed response [7].

There are currently no validated predictive biomarkers of the effectiveness of capecitabine treatment, and only four variants of the *DPYD* gene, which encodes for dihydropyrimidine dehydrogenase (DPD), the rate-limiting enzyme for FP metabolism, are clinically relevant in relation to their influence on the presence of severe toxicity during capecitabine therapy (rs3918290 (DPYD*2A), rs55886062 (DPYD*13), rs67376798, and rs75017182-rs56038477 (HapB3)) [8]. However, the genotyping of these four variants only prevents 20–30% of severe toxicity events associated with FPs. It seems that the remaining 70–80% of toxicities may be due to other genetic variants in *DPYD* or other genes involved in the PK and PD of capecitabine [9]. Therefore, the study of potential predictive genetic biomarkers of effectiveness and toxicity of capecitabine-based therapy is still necessary.

**Figure 1:**
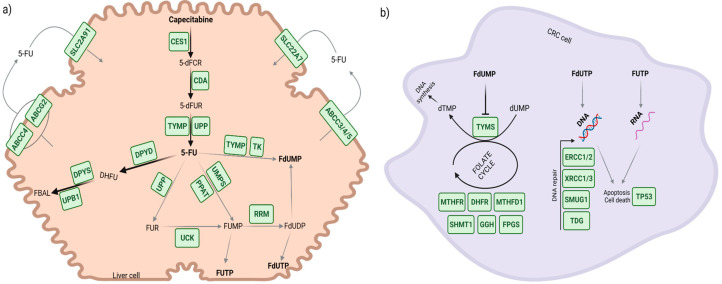
Main genes involved in the pharmacokinetic and pharmacodynamic pathways of capecitabine. (**a**) Main genes involved in capecitabine pharmacokinetics: bioactivation, metabolism, and transport. (**b**) Main genes involved in capecitabine pharmacodynamics: therapeutic targets, folate cycle, and DNA repair.

Several genes are involved in the PK of capecitabine. They can be grouped according to the bioactivation, catabolism, and transport processes of the drug (Figure 1a). The bioactivation of capecitabine comprises three steps. In the liver, capecitabine is catalyzed mainly by the carboxylesterase 1 (CES1) enzyme to form 5′-deoxy-5-fluorocytidine (5′-dFCR). 5′-dFCR is subsequently metabolized by the enzyme cytidine deaminase (CDA) from which 5′-deoxy-5-fluorouridine (5′-dFUR) is obtained. This metabolite is finally catalyzed to the active principle 5-FU by the enzymatic action of thymidine phosphorylase (TP) or uridine phosphorylase (UPP). TP is expressed in both liver and tumor tissue, but its expression in the latter is greater [10,11]. There are many 5-FU catabolism routes, some of which lead to the formation of inactive metabolites and others to the production of metabolites with pharmacological activity. As mentioned previously, the rate-limiting step of 5-FU catabolism is mediated by DPD, an enzyme that transforms it into dihydrofluorouracil (DHFU), which is subsequently metabolized by the dihydropirimidinase (DPYS) and beta-ureidopropionase 1 (UPB1) enzymes to obtain fluoro-beta-alanine (FBAL), a metabolite excreted in the urine. There are three pharmacologically active metabolites of 5-FU: fluorodeoxyuridine monophosphate (FdUMP), fluorouridine triphosphate (FUTP), and fluorodeoxyuridine triphosphate (FdUTP). The conversion of 5-FU into its main active metabolite FdUMP can occur by several routes: (a) direct action of the TP and then thymidine kinase (TK) enzymes, (b) indirect action of the uridine monophosphate synthetase (UMPS) and amidophosphoribosyltransferase (ATase) enzymes, of which the latter is encoded by the phosphoribosyl pyrophosphate amidotransferase (PPAT) gene, or (c) by UPP and uridine–cytidine kinase (UCK) to form the intermediate metabolite fluorouridine diphosphate (FUDP), which is subsequently catalyzed via ribonucleotide reductase (RNR). FUDP can also be converted into FUTP and FdUTP. Transport of 5-FU is mediated by various proteins, including equilibrative nucleoside transporter 1 (ENT1), encoded by solute carrier family 29 member 1 (*SLC29A1*) gene, solute carrier family 22 member 7 (SLC22A7), ATP-binding cassette subfamily G member 2 (ABCG2), ATP-binding cassette subfamily C member 3 (ABCC3), ATP-binding cassette subfamily C member 4 (ABCC4), and ATP-binding cassette subfamily C member 5 (ABCC5) [12]. The ATP-binding cassette subfamily B member 1 (*ABCB1*) gene, also known as multidrug resistance 1 (MDR1), codes for P-glycoprotein (P-gp), a carrier protein that transports a great variety of substrates and plays a crucial role in maintaining intracellular levels of numerous antineoplastic agents [13]. Although capecitabine has not been clearly identified as a substrate of P-gp, its expression has been related to resistance to 5-FU in modified cell lines, so its potential influence on the outcomes of therapy cannot be ruled out [14].

As for the PD of capecitabine (Figure 1b), the FdUMP metabolite inhibits the action of thymidylate synthase (TS), an enzyme encoded by the *TYMS* gene that is crucial for the synthesis of pyrimidines and DNA. In turn, this action blocks the simultaneous conversion of 5,10-methylenetetrahydrofolate (5,10-MTHF) to dihydrofolate, a key component of the folate cycle. Furthermore, FUTP and FdUTP metabolites are directly incorporated into RNA and DNA, respectively, causing direct damage to genetic material and consequently cell death. Therefore, as well as *TYMS*, the genes involved in (a) the folate cycle (methylenetetrahydrofolate reductase (*MTHFR*), dihydrofolate reductase (*DHFR*), methylenetetrahydrofolate dehydrogenase (*MTHFD1*), serine hydroxymethyltransferase 1 (*SHMT1*), gamma-glutamyl hydrolase (*GGH*), and folylpolyglutamate synthase (*FPGS*)), (b) DNA repair (ERCC excision repair 1 and 2 (*ERCC1*, *ERCC2*), single-strand-selective monofunctional uracil-DNA glycosylase 1 (*SMUG1*), thymine DNA glycosylase (*TDG*), X-ray repair cross complementing 1 and 3 (*XRCC1*, *XRCC3*), and (c) the cell cycle (tumor protein P53 (*TP53*)), are of particular interest in the PD of capecitabine [12].

The objective of this study was to systematically review the literature published in the last decade on the influence of single-nucleotide polymorphisms (SNPs) in the main genes involved in the PK and PD of capecitabine on the effectiveness and safety of antineoplastic therapy in patients with CRC.

## 2. Materials and Methods

### 2.1. Search Strategy

The reporting of this systematic review was guided by the standards of the Preferred Reporting Items for Systematic Reviews and Meta-Analyses (PRISMA) Statement [15]. A literature search for studies that evaluated associations between SNPs in genes involved in the PK and PD of capecitabine with treatment effectiveness and toxicity was performed in PubMed and Scopus databases until 30 December 2022. The search strategy consisted of a combination of the following terms connected by the boolean operator AND (Supplementary Table S1). Gene term: (Gene abbreviation OR Gene full name) for *CES1*, *CES1P1*, *CES2*, *CDA*, *DPYD*, *DPYS*, *PPAT*, *RRM2*, *RRM1*, *TK1*, *TYMP*, *UCK1*, *UCK2*, *UMPS*, *UPP1*, *UPP2*, *UPB1*, *ABCB1*, *ABCC3*, *ABCC4*, *ABCC5*, *ABCG2*, *ABCB1*, *SLC22A7*, *SLC29A1*, *TYMS*, *ENOSF1*, *MTHFR*, *DHFR*, *MTHFD1*, *SHMT1*, *GGH*, *FPGS*, *ERCC2*, *ERCC1*, *SMUG1*, *TDG*, *XRCC3*, *XRCC1*, and *TP53* genes. Drug term: Capecitabine. Disease term: (colon OR colonic OR colorectal OR rectal) AND (neoplasm OR cancer OR carcinoma OR malignant OR malignancy).

Duplicate articles were deleted, and the remaining articles were analyzed by title and abstract. Those that met the inclusion criteria were evaluated by reading the full text. Search and study selection was performed independently by two researchers. In the event of discrepancies in the comparison of results, a third researcher was consulted.

### 2.2. Eligibility Criteria

Studies were selected if they complied with the following inclusion criteria: patients treated solely with therapeutic regimens based on capecitabine (as monotherapy or in combination with other antineoplastic agents or RT) for all types of treatment (neoadjuvant, adjuvant, palliative); patients genotyped for SNPs in *CES1*, *CES1P1*, *CES2*, *CDA*, *DPYD*, *DPYS*, *PPAT*, *RRM2*, *RRM1*, *TK1*, *TYMP*, *UCK1*, *UCK2*, *UMPS*, *UPP1*, *UPP2*, *UPB1*, *ABCB1*, *ABCC3*, *ABCC4*, *ABCC5*, *ABCG2*, *ABCB1*, *SLC22A7*, *SLC29A1*, *TYMS*, *ENOSF1*, *MTHFR*, *DHFR*, *MTHFD1*, *SHMT1*, *GGH*, *FPGS*, *ERCC2*, *ERCC1*, *SMUG1*, *TDG*, *XRCC3*, *XRCC1*, or *TP53* genes; evaluation of therapy effectiveness using RECIST criteria or Dworak classification (for tumor response) or survival and toxicity analysis (using the Common Terminology Criteria for Adverse Events (CTCAE)); full-text availability; original design type: randomized clinical trial, non-randomized clinical trial or experimental study, cohort study (prospective or retrospective), or case-control study; published in the last 10 years in English, Spanish, Portuguese, or German. If a cohort (or part of a cohort) was described in more than one study, the most recent or extensive study was selected. Conversely, studies that presented the same cohort (or part of the same cohort) but evaluated different genes of interest were included. Exclusion criteria were extraction of genetic material from tumor tissue and studies based solely on haplotypes, mutation studies, gene expression studies. Case report/case series articles, editorials, letters to the editor, clinical guidelines, and reviews were excluded.

### 2.3. Data Extraction

Extracted data included study design, clinical data collection, ethnicity, study size, mean age, women percentage, CRC stage, treatment regimen and setting, genes of interest investigated, type of outcome studied, and statistical measures of outcome. The extraction of data from the articles included was performed and reviewed by two researchers. The terminology employed by each author of the studies included was used to record outcome measures. The measure of association was calculated when it was not present in a study only if the information necessary for its determination was available. If any of the selected studies presented results for genetic variants other than SNPs, only the results related to the latter were collected.

### 2.4. Quality Assessment

To assess the quality of the included studies, a descriptive analysis was performed by two researchers evaluating nine items obtained from the Strengthening the Reporting of Genetic Association studies (STREGA) criteria [16]. In case of any discrepancies, a third researcher was consulted. The items analyzed covered the following subjects: (a) laboratory methods, (b) number of samples genotyped and genotyping success rate, (c) population stratification methodology, (d) genotype or haplotype inference methods, (e) Hardy–Weinberg equilibrium (HWE), and (f) indication of the novelty of the genetic association study. A value of 1 was assigned if the study complied with the item (Y), 0.5 if the Item was incomplete (I), and 0 if it was not complied with (N). A total score was calculated for each study by adding all the scores for the items assessed (range 0 to 9). Total score was expressed as a percentage (points obtained/maximum points × 100) to classify the studies as high (>80%), moderate (50–80%), or low (<50%) quality, as done previously [17,18,19].

## 3. Results

### 3.1. Search Results

**Figure 2:**
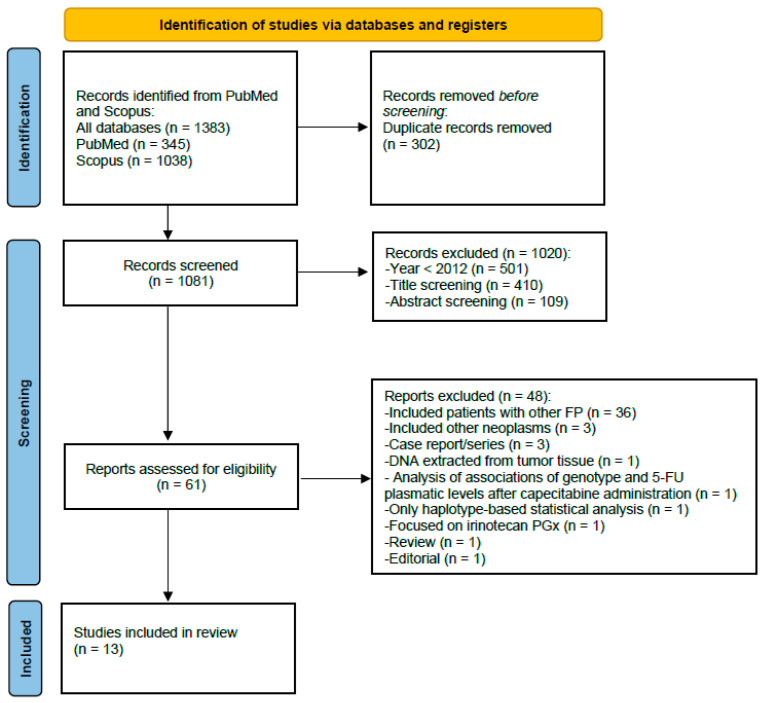
Preferred Reporting Items for Systematic Reviews and Meta-Analyses (PRISMA) flow diagram. FPs: fluoropyrimidines; PGx: pharmacogenetics.

The initial search delivered 1383 studies (Figure 2). After deletion of duplicates (*n* = 302) and removal of studies that did not comply with the inclusion criteria in the screening of titles and abstracts (*n* = 1020), 61 records were preselected for full-text review. Of these, 36 were eliminated because they included patients treated with other FPs (5-FU or S-1), 1 because DNA was obtained from tumor tissue, 1 because variant analysis focused on irinotecan-based therapy, 1 because its statistical analysis focused on association of genetic polymorphisms with plasma 5-FU levels, 1 because the association analysis was performed by haplotypes and not by single variants, 3 because patients with neoplasms other than CRC were included, and 5 for not complying with the study type for selection (3 case reports/case series, 1 editorial, 1 review). Finally, 13 studies were included in this review.

### 3.2. Study Characteristics

The included studies were published between 2013 and 2021 [20,21,22,23,24,25,26,27,28,29,30,31,32]. Most were cohort studies [20,21,22,23,24,26,28,29,30,31]. Clinical data collection was mainly prospective [20,21,23,27,28,29,30]. Eight studies were conducted in populations of European ancestry [20,21,23,24,25,26,29,31], two in the Indian population [28,30], and one in patients of Asian origin [32]. In two studies, the ethnicity of the study population was not specified [22,27]. Regarding the treatment regimens studied, five studies evaluated patients under treatment with capecitabine in combination with other neoplastic agents [23,24,28,29,30], three with capecitabine as monotherapy [20,27,31], and five with capecitabine as monotherapy and in combination [21,22,25,26,32]. Five studies only investigated capecitabine PD-related genes [20,24,27,29,32], two only investigated PK-related genes [23,28], and six studied genes related to both [21,22,25,26,30,31]. Eight studies evaluated the influence of SNPs on the incidence and severity of capecitabine-induced toxicity [21,22,23,25,26,28,31,32], three assessed the influence of SNPs on the effectiveness of capecitabine-based therapy [24,27,30], and two evaluate the influence of SNPs on both toxicity and effectiveness [20,29]. The main characteristics of the included studies are shown in Table 1.

### 3.3. SNPs Associated with Capecitabine-Induced Toxicity

Genes, SNPs, and genotypes associated with capecitabine-induced toxicity are summarized in Table 2.

#### 3.3.1. Cytidine Deaminase Gene (CDA)

For the *CDA* gene, two studies included in this review reported significant associations with severe toxicity in capecitabine-based therapy [22,25]. A study conducted in Spain found that the AA genotype of the *CDA* rs2072671 SNP (c.79A>C; missense; p.Lys27Gln) was associated with a greater risk of severe overall toxicity (grade 3–4) in 239 patients with CRC (stages I–IV) treated with various capecitabine-based therapeutic regimens (OR = 1.84; 95% CI = 1.06–3.18; *p* = 0.029 for AA vs. AC/CC) [22]. In line with these results, Pellicer et al. (European ancestry; Spain) found a protective effect of the C allele of this SNP against severe overall toxicity in 301 patients with CRC (stages I–IV) under treatment with capecitabine-based therapeutic regimens (OR = 0.5; 95% CI = 0.30–0.83; *p* = 0.007 for AC/CC vs. AA) [25]. They also found a significant association between this genotype and the presence of severe hand–foot syndrome (HFS) (OR = 0.27; 95% CI = 0.10–0.71; *p* = 0.008 for AC/CC vs. AA) [25]. Moreover, they reported that the T allele of the *CDA* rs1048977 SNP (c.435C>T; synonymous; p.Thr145Thr) was associated with the presence of severe hyperbilirubinemia (OR = 8.62; 95% CI = 1.05–70.24; *p* = 0.044 for CT/TT vs. CC) [25].

#### 3.3.2. Dihydropyrimidine Dehydrogenase Gene (DPYD)

Six studies included in this review evaluated the association of SNPs in the *DPYD* gene with capecitabine-based therapy toxicity [21,23,25,26,28,31]. Of these, only two studies reported results in relation to the four relevant SNPs in current clinical practice: rs3918290 (c.IVS14+1G>A; splice donor), rs55886062 (1679T>G; missense; p.Ile560Ser), rs67376798 (c.2846A>T; missense; p.Asp949Val), and rs56038477 (c.1236G>A; synonymous; p.Glu412Glu) [21,31]. Rosmarin et al. (European ancestry; UK) analyzed the association of 1456 SNPs with toxicity in 940 patients with CRC (stages II–III) from the QUSAR2 trial (Phase III randomized controlled trial of adjuvant capecitabine ± bevacizumab), using a strict Bonferroni-corrected *p*-value threshold of 3.43 × 10^–5^ (0.05/1456) [21]. In this study, none of the clinically relevant SNPs reached a significant association. In turn, Puerta-García et al. (European ancestry; Spain), who also defined a Bonferroni-corrected *p* value of 0.010 (0.05/5), found no significant association between the AT genotype of the rs67376798 SNP and the presence of severe overall toxicity (*p* = 0.287) or any toxicity subtype (gastrointestinal, cardiovascular, asthenia, cutaneous, and respiratory) in 84 patients with CRC (stages I–IV) receiving first-line treatment with adjuvant capecitabine (*p* > 0.010) [31]. 

The association of other SNPs in the *DPYD* gene with capecitabine-based therapy toxicity has also been investigated [21,23,25,26,28,31]. Pellicer et al. (European ancestry; Spain) reported a significant association between the *DPYD* rs1801160 SNP (c.2194G>A; missense; p.Val732Ile) and overall toxicity (grade 2–4) in 319 patients with CRC (stages I–IV) treated with capecitabine-based therapeutic regimes (OR = 2.11; 95% CI = 1.08–4.13; *p* = 0.029 for AG/AA vs. GG) [26]. In contrast, neither the previously mentioned study by Rosmarin et al. (European ancestry; UK) nor Varma et al. (Asian; India) in 141 patients with CRC (stages II–IV) treated with capecitabine + oxaliplatin (CAPOX), found a significant association between the A allele of this SNP and the presence of toxicity [21,28]. 

As for the *DPYD* rs1801265 SNP (c.85T>C; missense; p.Cys29Arg), Varma et al. (Asian; India) reported that patients carrying the C allele showed an increased risk of thrombocytopenia (OR = 2.40; 95% CI = 1.18–5.10; *p* = 0.010 for CT/CC vs. TT), diarrhea (OR = 2.70; 95% CI = 1.80–4.00; *p* = 0.040 for CT/CC vs. TT), and HFS (OR = 2.30; 95% CI = 1.80–4.00; *p* = 0.020 for CT/CC vs. TT) during CAPOX treatment [28]. On the other hand, Rosmarin et al. (European ancestry; UK) found no significant association between the C allele of this SNP and the presence of severe overall toxicity (OR = 0.76; 95% CI = 0.58–1.01; *p* = 0.078 for CT/CC vs. TT) [21]. 

Regarding the *DPYD* rs2297595 SNP (c.496A>G; missense; p.Met166Val), Falvella et al. (European ancestry; Italy) reported a significant association between the AG genotype and the presence of severe overall toxicity in 64 patients with metastatic CRC treated with capecitabine + oxaliplatin + irinotecan (CAPOXIRI) in combination with bevacizumab or cetuximab (OR = 5.94; 95% CI = 1.29–27.22; *p* = 0.022 for AG vs. AA) [23]. In contrast, the study by Rosmarin et al. (European ancestry; UK) did not report a significant association between carriers of the G allele and severe toxicity (OR = 0.92; 95% CI = 0.64–1.31; *p* = 0.010 for AG/GG vs. AA) [21]. 

In the case of the *DPYD* rs12022243 SNP (c.1906-14763G>A; intron) in linkage disequilibrium (LD) with *DPYD* rs7548189 (c.1906-19696G>T; intron), Rosmarin et al. (European ancestry; UK) reported a significant association with diarrhea (OR = 1.79; 95% CI = 1.54–2.05; *p* = 9.86 × 10^–6^ for A vs. G and OR = 1.76; 95% CI = 1.50–2.02; *p* = 1.72 × 10^–5^ for T vs. G respectively). The rs12132152 SNP (97523004G>A) in LD with rs76387818 (g.97539400G>A), an intergenic SNP located 22 kb downstream of *DPYD* gene was also associated with severe overall toxicity (OR = 3.83; 95% CI = 3.26–4.40; *p* = 4.31 × 10^–6^ and OR = 4.05; 95% CI = 3.47–4.62; *p* = 2.11 × 10^–6^, respectively, both for A vs. G) and severe HFS (OR = 6.12; 95% CI = 5.58–6.76; *p* = 3.29 × 10^–8^ and OR = 6.44; 95% CI = 5.79–7.09; *p* = 1.75 × 10^–8^, respectively, both for A vs. G) [21]. 

Regarding the *DPYD* rs17376848 SNP (1896T>C; synonymous; p.Phe632Phe), Falvella et al. (European ancestry; Italy) reported that patients carrying the CT genotype showed an increased risk of severe overall toxicity (OR = 14.53; 95% CI = 1.36–155.20; *p* = 0.027 for CT vs. TT) [23]. In contrast, the study by Puerta-García et al. (European ancestry; Spain) reported no significant association between the CT genotype and severe overall toxicity (OR = 2.51; 95% CI = 0.03–202.96; *p* = 0.494 for CT vs. TT) or any other toxicity subtype [31]. 

Regarding the *DPYD* rs12119882 SNP (c.680+2545T>C; intron), Pellicer et al. (European ancestry; Spain) found, in 301 patients with CRC (stages I–IV) treated with capecitabine-based regimens, that patients carrying the C allele had an increased risk of severe hyperbilirubinemia (OR = 4.86; 95% CI = 1.16–20.38; *p* = 0.031 for CT/CC vs. TT) [25]. 

Finally, Rosmarin et al. (European ancestry; UK) also evaluated the influence of the *DPYD* rs1801158 (c.1601G>A; missense; p.Ser534Asn), rs1801159 (c.1627A>G; missense; p.Ile543Val), and rs45589337 (c.775A>G; missense; p.Lys259Glu) SNPs on the toxicity of capecitabine-based therapy, without significant results [21].

#### 3.3.3. Uridine Monophosphate Synthetase Gene (UMPS)

Only one study included in the review reported significant associations between SNPs of the *UMPS* gene and capecitabine-based therapy toxicity [25]. Pellicer et al. (European ancestry; Spain) reported that the C allele of the *UMPS* rs4678145 SNP (c.156+607G>C; intron) was associated with a higher risk of severe asthenia (OR = 4.54; 95% CI = 1.55–13.24; *p* = 0.006 for CG/CC vs. GG), and that the C allele of the *UMPS* rs2279199 SNP (c.-67T>C; 2KB upstream) showed a protective effect against severe nausea and vomiting (OR = 0.21; 95% CI = 0.04–0.90; *p* = 0.036 for CT/CC vs. TT) in 301 patients with CRC (stages I–IV) treated with capecitabine-based therapeutic regimens [25].

#### 3.3.4. Solute Carrier Family 22 Member 7 Gene (SLC22A7)

Pellicer et al. (European ancestry; Spain) studied the influence of SNPs in the *SLC22A7* gene on severe toxicity in 301 patients with CRC (stages I–IV) treated with capecitabine-based regimens and found that the TT genotype of the *SLC22A7* rs2270860 SNP (1269C>T; synonymous; p.Ser423Ser) was associated with an increased risk of severe cutaneous toxicity (OR = 17.08; 95% CI = 1.71–170.26; *p* = 0.016 for TT vs. CT/CC) [25]. They also reported a protective effect of the G allele of the *SLC22A7* rs4149178 SNP (1592+206A>G, intron) against severe diarrhea (OR = 0.34; 95% CI = 0.12–0.92; *p* = 0.034 for AG/GG vs. AA) [25].

#### 3.3.5. ATP-Binding Cassette Subfamily B Member 1 Gene (ABCB1)

Regarding the *ABCB1* gene, Puerta-García et al. (European ancestry; Spain) found no significant associations in univariate analysis performed with Bonferroni correction (*p* > 0.01) for the CC genotype of the *ABCB1* rs1128503 SNP (c.1236T>C; synonymous; p.Gly412Gly) with severe overall toxicity, or any other toxicity subtype, in 84 patients with CRC (stages I–IV) receiving first-line treatment with adjuvant capecitabine [31].

#### 3.3.6. Thymidylate Synthetase Gene (TYMS) and Enolase Superfamily Member 1 (ENOSF1)

Four studies included in this review reported association of SNPs in regions of the *TYMS* gene, and its adjacent gene *ENOSF1*, with capecitabine-based therapy toxicity [21,22,25,32]. Pellicer et al. (European ancestry; Spain) reported that the CC genotype of the *TYMS* rs2853741 SNP (c.-391T>C; 2KB upstream) showed a protective effect against severe diarrhea in 301 patients with CRC (stages I–IV) undergoing treatment with capecitabine-based regimens (OR = 0.31; 95% CI = 0.13–0.74; *p* = 0.008 for CC vs. CT/TT) [25]. Dong et al. (Asian; China) reported a significant association between the *TYMS* rs3786362 SNP (c.381A>G; synonymous; Glu127Glu) with grade 2–3 HFS in 288 patients with CRC (stages I–IV) treated with capecitabine-based regimens (OR = 0.38; 95% CI = 0.21–0.70; *p* = 1.89 × 10^–3^ for AA vs. AG vs. GG) [32]. The two aforementioned studies reported a significant association of the rs699517 SNP (c.*19C>T; 3′UTR/noncoding transcript) in the *TYMS*/*ENOSF1* region with capecitabine-based therapy toxicity [25,32]. Pellicer et al. (European ancestry; Spain) reported that carriers of the TT genotype showed a higher risk of nausea/vomiting and severe diarrhea (OR = 7.93; 95% CI = 1.51–41.63; *p* = 0.014 and OR = 128.82; 95% CI = 4.16–3988.96; *p* = 0.006, respectively, both for TT vs. CT/CC) and that the T allele also showed a protective effect against severe asthenia (OR = 0.24; 95% CI = 0.07–0.81; *p* = 0.021 for CT/TT vs. CC) [25]. Dong et al. (Asian; China), in turn, reported a significant association between this SNP and grade 2–3 HFS (OR = 2.12; 95% CI = 1.39–3.24; *p* = 4.62 × 10^–4^ for CC vs. CT vs. TT) [32]. This study also reported a significant association of the rs2790 SNP (c.*89A>G; 3′UTR/intron) in the *TYMS/ENOSF1* region with grade 2–3 HFS (OR = 0.58; 95% CI = 0.39–0.87; *p* = 8.80 × 10^–3^ for AA vs. AG vs. GG) [32]. 

Moreover, two of the studies included in this review reported significant associations between SNPs of the *ENOSF1* gene and toxicity during capecitabine-based therapy [21,22]. Rosmarin et al. (European ancestry; UK) reported a significant association of the G allele of the *ENOSF1* rs2612091 SNP (c.496-227G>A; intron) with overall toxicity and severe HFS in 940 patients with CRC (stages II–III) treated with capecitabine ± bevacizumab (OR = 1.59; 95% CI = 1.39–1.79; *p* = 5.28 × 10^–6^ and OR = 1.57; *p* = 2.94 × 10^–6^, respectively, both for G vs. A) [21]. In turn, García-González et al. (Spain) found that the GG genotype of this SNP was significantly associated with grade 2–4 HFS in 239 patients with CRC (stages I–IV) treated with capecitabine-based regimens (OR = 2.28; 95% CI = 1.10–4.76; *p* = 0.027 for GG vs. GA/AA) [22]. Rosmarin et al. (European ancestry; UK) also reported a significant association with another SNP of the *ENOSF1* gene. Patients carrying the A allele of the *ENOSF1* rs2741171 SNP (c.63+5783A>G; intron) showed an increased risk of overall toxicity and severe HFS (OR = 1.60; 95% CI = 1.39–1.80; *p* = 6.64 × 10^–6^ and OR = 1.74; 95% CI = 1.51–1.97; *p* = 1.64 × 10^–6^ respectively, both for A vs. G) [21].

#### 3.3.7. Methylenetetrahydrofolate Reductase Gene (MTHFR)

Two of the studies included in this review reported results in relation to the influence of SNPs in the *MTHFR* gene on capecitabine therapy toxicity. Only Puerta-García et al. (European ancestry; Spain) reported a significant association of the TT genotype of the *MTHFR* rs1801133 SNP (c.665C>T; missense; p.Ala222Val) with the risk of asthenia in 84 patients with CRC (stages I–IV) receiving first-line treatment with adjuvant capecitabine (OR = 9.30; 95% CI = 1.36–106.8; *p* = 0.009 for TT vs. CT/CC) [31]. No significant association was reported between the *MTHFR* rs1801131 SNP (1286A>C; missense; p.Glu429Ala) and toxicity in CRC patients treated with capecitabine [20,31].

### 3.4. Gene Variants Associated with Capecitabine Effectiveness

The genes, SNPs, and genotypes associated with capecitabine effectiveness are summarized in Table 3.

#### 3.4.1. ATP-Binding Cassette Subfamily B Member 1 Gene (ABCB1)

Varma et al. (Asian; India) investigated the influence of SNPs in the *ABCB1* gene on the response to adjuvant treatment with CAPOX in 145 CRC patients (stages II–IV). This study found no significant association between the *ABCB1* rs1128503 and rs1045642 SNPs (c.3435T>C; synonymous; p.Ile1145Ile) and treatment response [30].

#### 3.4.2. ERCC Excision Repair 1 (ERCC1)

Three studies included in this review reported results with respect to the influence of SNPs in the *ERCC1* gene on the effectiveness of capecitabine-based treatment [24,29,30]. Sebio et al. (European ancestry; Spain) reported a significant association of the *ERCC1* rs11615 SNP with response to neoadjuvant capecitabine/RT (*p* = 0.023) in 84 patients with CRC (stages II–III) [24]. In contrast, Varma et al. (Asian; India) found no significant association between the C allele of the *ERCC1* rs11615 SNP and response to CAPOX adjuvant therapy in 145 CRC patients (stages II–III) (OR = 0.50; 95% CI = 0.10–2.00; *p* = 0.300 for CT/CC vs. TT) [30]. Moreover, Boige et al. (European ancestry; France) reported that the G allele of the *ERCC1* rs10412761 SNP (g.45908461A>G) was associated with a decreased response to capecitabine/RT or CAPOX/RT neoadjuvant therapy in 316 CRC patients (stages II–III) (OR = 0.57; 95% CI = 0.34–0.98; *p* = 0.042 for AG/GG vs. AA) [29]. This study found no significant relationship between this SNP and overall survival (OS) (HR = 1.47; 95% CI = 0.85–2.56; *p* = 0.160 for AG/GG vs. AA) [29].

#### 3.4.3. ERCC Excision Repair 2 (ERCC2)

Two studies included in this review provided results related to the association of SNPs in the *ERCC2* gene with the effectiveness of capecitabine-basedtherapy [29,30]. Boige et al. (European ancestry; France) found a significant association of the T allele of the *ERCC2* rs1799787 SNP (c.1832-70C>T, intronic) with a decreased response to capecitabine/RT or CAPOX/RT neoadjuvant treatment in 316 CRC patients (stages II–III) (OR = 0.55; 95% CI = 0.33–0.93; *p* = 0.027 for CT/TT vs. CC) [29]. This study found no significant association of the *ERCC2* rs13181 SNP (c.2251A>C; stop gained; p.Lys751Ter) with OS (HR = 0.73; 95% CI = 0.43–1.22; *p* = 0.235 for AC/CC vs. AA) [29]. Similarly, Varma et al. (Asian; India) found no association of this SNP with response to adjuvant CAPOX treatment in 145 patients with CRC (stages II–III) (OR = 0.80; 95% CI = 0.10–4.00; *p* = 0.500) [30].

#### 3.4.4. Methylenetetrahydrofolate Reductase Gene (MTHFR)

None of the studies that investigated the influence of SNPs in the *MTHFR* gene on treatment effectiveness reported statistically significant results [20,27,29].

### 3.5. Quality Assessment

The quality score assigned to each included study is available in Supplementary Table S2. Quality scores ranged between 33.33–72.22%. Most of the studies (7/13; 53.85%) had a moderate level of quality. Six studies (46.15%) fully reported the genotyping methods used. The seven studies (53.85%) that were considered incomplete did not provide information on DNA storage conditions or genotyping platforms used. All studies (100%) indicated whether their research reported new associations, replicated previous studies, or both. Nine studies (69.23%) reported both the number of samples to be genotyped and those that were successfully genotyped and considered HWE in the analysis. A substantial proportion of the included studies did not mention call rates and error rates (9/13; 69.23%), the center where genotyping was performed (12/13; 92.31%) or did not mention whether or not genotype or haplotype inference was performed (8/13; 61.54%).

## 4. Discussion

This systematic review identified numerous SNPs in genes involved in the PK and PD of capecitabine that may influence the toxicity or effectiveness of antineoplastic therapy in CRC patients. Genes involved in bioactivation, metabolism, transport, or mechanism of action of capecitabine, DNA repair, and the folate cycle have been associated with toxicity, while genes involved in DNA repair have been significantly associated with therapy effectiveness.

*CDA* gene plays an important role in the bioactivation of capecitabine into 5-FU and in the detoxification of other antimetabolite elements such as gemcitabine, decitabine, and cytarabine [33]. Studies included in this review reported that *CDA* rs2072671 and rs1048977 SNPs were significantly associated with overall toxicity, HFS, and severe hyperbilirubinemia [22,25]. Both these SNPs have been associated with altered PK, enzyme activity, and exposure to drugs metabolized by CDA [34,35]. It has been reported that the C allele of the *CDA* rs2072671 SNP has a detrimental effect on enzyme activity, which would hypothetically lead to a decrease in capecitabine activation, and consequently less exposure to 5-FU [36]. However, other studies provide contradictory information and indicate that the resulting catalytic activity depends on the substrate analyzed [33,37,38]. Studies conducted in patients of Asian (China) and European (Switzerland, The Netherlands) ancestry with gastric neoplasms treated with capecitabine-based regimens have not reported a significant association between this SNP and severe toxicity [39,40]. In line with the results of this review, two meta-analyses, in 1093 and 623 patients of mixed (international) ethnicity with pancreatic and non-small-cell lung cancer (NSCLC) treated with gemcitabine, found a significant association between the C allele of the *CDA* rs2072671 SNP and the presence of hematological toxicity [34,38]. Both studies highlighted this SNP as a potential predictive biomarker of antineoplastic therapy toxicity [34,38]. 

There are no other studies that have been conducted in patients treated with capecitabine for the *CDA* rs1048977 SNP. However, a narrative review reported that of a total of two included studies evaluating the association of this SNP with gemcitabine toxicity, only one reported that pancreatic cancer patients of mixed ethnicity (USA) carrying the T allele were significantly associated with neutropenia [34]. In turn, recent studies in patients with solid tumors of the pancreas, bladder, and lung in European (Poland) and Asian (China) populations have also reported a significant association of the T allele of this SNP with gemcitabine-related toxicity [41,42]. The DPD enzyme is the first and rate-limiting step in the catabolism of 5-FU, converting it into the metabolite DHFU. *DPYD* is the only gene that currently has SNP-validated biomarkers of FP toxicity in everyday practice [8]. No studies reporting significant results with regard to the association of the clinically relevant *DPYD* SNPs rs3918290, rs55886062, rs67376798, and rs75017182/rs56038477 with capecitabine-based treatment toxicity were included in this review. This may be due to (a) the fact that these are the first variants studied in relation to FP-associated toxicity, and therefore most of the studies focusing mainly on these SNPs could have been published more than 10 years ago, (b) the extremely low frequency of these SNPs in the overall population, requiring a larger sample size than the studies included in this review, and (c) the fact that the great majority of studies of these SNPs have been conducted in patients with various solid neoplasms treated with 5-FU based regimens or without distinction of FP agent [8,43,44]. 

Over the last decade, there has been a notable increase in the study of the influence of SNPs other than the four clinically relevant variants in the *DPYD* gene, which have a relatively higher frequency in the overall population, on capecitabine-based therapy toxicity. This review included five studies that reported contradictory results regarding the association of various SNPs in the *DPYD* gene with capecitabine toxicity [21,23,25,26,28,31]. These results agree with studies conducted in patients with other cancers or treated with other FPs [45,46,47,48,49,50,51,52]. A meta-analysis of six studies in 6119 European ancestry (international) patients with solid neoplasms (gastrointestinal, breast, pancreas, bile duct, among others) treated with FPs reported that the *DPYD* rs1801160 SNP was associated with an increased risk of toxicity, indicating that this SNP should be included in clinical practice [45]. In turn, a study in 503 CRC patients of European ancestry (Croatia) treated with FPs reported that patients carrying the *DPYD* rs1801160 and rs2297595 SNPs showed, respectively, a tendency towards and a significant association with severe adverse events. This study also mentioned that the inclusion of these SNPs in clinical practice should be considered [46]. Similar findings are reported by a study in 508 patients of European ancestry (Italy) with CRC (stages II–III) treated with FP. This study found a significant association between the *DPYD* rs1801160 and rs2297595 SNPs and severe adverse events during therapy [47]. Two recent studies in patients with gastrointestinal neoplasms (*n* = 80 and 93), of Jordanian (Jordan) and Latin American (Chile) origin, reported a significant association between the *DPYD* rs1801265 SNP and adverse events during FP therapy [49,50]. However, two other studies conducted in patients with gastrointestinal neoplasms (*n* = 503 and 113), of European and African American origin (Croatia, USA), found no significant association between this SNP and FP severe toxicity [46,51]. The differences in these findings may be due to several factors, such as sample size and ethnicity of the population being investigated. A similar situation occurred with the *DPYD* rs17376848 SNP regarding sample size. A previous study in 64 European patients (Switzerland) with metastatic gastrointestinal carcinomas treated with capecitabine-based therapeutic regimens reported a significant association between this SNP and diarrhea and HFS [52]. However, recent studies with larger sample sizes (*n* = 508 and 1254 patients, respectively) in European populations (Italy) with solid stomach, colon, and breast tumors did not demonstrate such association [47,48].

UMPS is an enzyme that metabolizes 5-FU into other metabolites with cytotoxic activity and is considered to be a major regulator of the cytotoxic effects of 5-FU [53]. One study included in this review reported an association of the *UMPS* rs4678145 and rs2279199 SNPs with asthenia and severe nausea/vomiting in CRC patients treated with capecitabine-based regimens [25]. However, a similar study in 338 Asian patients (China) with gastrointestinal cancers treated with capecitabine found no significant association between the *UMPS* rs4678145 and rs2279199 SNPs and severe toxicity [54].

*SLC22A7* is the gene that encodes the organic anion transporter 2 (OAT2). This protein is responsible for transporting 5-FU within cells [55]. One study included in this review reported an association of the *SLC22A7* LD SNPs rs2270860 and rs4149178 with cutaneous toxicity and severe diarrhea in CRC patients treated with capecitabine-based regimens [25]. Evidence on SNPs in this gene and their relationship with transporter activity is limited, and no significant association has been found between these variants and OAT2 hepatic expression [55]. There are no other studies that evaluate the influence of these SNPs on capecitabine-based treatment toxicity. However, a study in 344 pediatric cancer patients (mostly with leukemias and lymphomas) of mixed ethnicity (Canada) described an association between the G allele of the *SLC22A7* rs4149178 SNP and a lower risk of cardiotoxicity during anthracycline treatment [56].

TS is a key enzyme for DNA biosynthesis and is the main therapeutic target of 5-FU [57]. ENOSF1 is an enzyme with numerous isoforms. While one isoform exhibits catalytic activity, others seem to have a regulatory role on TS activity [58]. *ENOSF1* and *TYMS* genes partially overlap and are transcribed in opposite directions [59]. Four studies included in this review reported the association of several SNPs in these genes (*ENOSF1* rs261091 and rs2741171, *TYMS/ENOSF1* rs699517 and rs2790, and *TYMS* rs2853741 and rs3786362) with the risk of toxicity during capecitabine treatment, especially HFS [21,22,25,32]. A meta-analysis of 1912 patients of predominantly European ancestry (international) with gastrointestinal cancers reported that carriers of the G allele of the *ENOSF1* rs2612091 SNP showed a higher risk of HFS during FP treatment. This study highlighted the essential role of *ENOSF1* and of other variants in *TYMS* (6bp-indel and 28bp-repeat) in HFS development during FP treatment [59]. Another study in 342 Asian patients (China) with metastatic breast cancer under capecitabine-based therapy reported that patients carrying the CT genotype of the *TYMS* rs2853741 SNP had 2.25 times more risk of HFS [60].

MTHFR is a critical enzyme in the folate cycle and plays an important role in the PD of FPs. This enzyme catalyzes the irreversible conversion of 5,10-methylenetetrahydrofolate (5,10-MTHF) to 5-methyltetrahydrofolate, reducing the amount of 5,10-MTHF available. 5,10-MTHF is essential for the formation of a ternary complex with the active metabolite of 5-FU, FdUMP, and the TS enzyme, which results in the inhibition of the enzyme [61]. It has been suggested that MTHFR activity may be a crucial factor for predicting FP response and toxicity [61,62]. The *MTHFR* rs1801133 SNP results in decreased MTHFR activity leading to an increased intracellular concentration of 5,10-MTHF, which could enhance the formation of the 5,10-MTHF/FdUMP/TS ternary complex, increasing the risk of FP toxicity [63]. Only one study included in this review reported a significant association between the *MTHFR* rs1801133 SNP and toxicity during capecitabine therapy [31]. A study conducted in 50 patients of Latin American origin (Costa Rica) with metastatic CRC treated with FP-based chemotherapy reported that carriers of the T allele showed a higher risk of hematological, neurological, and HFS toxicity [62]. A meta-analysis of 20 publications revealed that in 1635 lung cancer patients of Asian (China) and European (Spain) origin, carriers of the T allele of the *MTHFR* rs1801133 SNP showed a higher risk of hematological toxicity during oxaliplatin-based therapy [64].

*ERCC1* and *ERCC2* are nucleotide excision repair genes. They are part of the so-called DNA repair genes and play a key role in tumor response to chemotherapy-induced DNA damage [29]. ERCC1 protein is involved in the DNA damage incision process and ERCC2 in the damage unwinding process. Polymorphisms in *ERCC1* and *ERCC2* could alter the ability to repair DNA, thereby affecting the response or survival of cancer patients [65]. Two studies included in this review reported a significant association of the *ERCC1* rs11615, *ERCC1* rs10412761, and *ERCC2* rs1799787 SNPs with response to neoadjuvant capecitabine-based chemoradiation in European ancestry patients (Spain, France) [24,29]. However, contradictory results have been reported for the *ERCC1* rs11615 SNP in patients of Asian origin (India) under adjuvant CAPOX treatment [30]. The most widely studied SNP of the *ERCC1* gene is rs11615 [66,67,68]. It has been reported that this SNP has a negative impact on mRNA expression level, which is related to a decrease in ERCC1 repair function, resulting in an improved response to cytotoxic treatment [69]. A meta-analysis of six studies in 1137 patients with osteosarcoma suggested that in one Asian population (China), the *ERCC1* rs11615 SNP is significantly associated with response to platinum-based chemotherapy, indicating that carriers of the C allele would benefit more from therapy [66]. In turn, a meta-analysis of 26 studies in 1401 patients of Asian and European ancestry (international) with NSCLC, revealed that the *ERCC1* rs11615 SNP was associated with overall response rate (ORR) [67]. In contrast, a meta-analysis of 22 studies including 2846 patients of Asian and European ancestry (international) with advanced CRC treated with FPs and platinum found no significant association between the *ERCC1* rs11615 SNP and ORR [68]. However, it did report a significant correlation between the T allele of this SNP and lower OS and progression-free survival (PFS). Notably, stratified analysis by ethnicity revealed that the T allele of this SNP was associated with worse survival profiles in patients of Asian origin, but with favorable prognostic outcomes in European ancestry patients. This finding reveals the high significance of ethnicity regarding genetic influence on therapy outcomes [68]. Concerning the *ERCC1* rs10412761 SNP, previous studies on populations of predominantly European ancestry (USA) with pancreatic and ovarian cancer treated with several therapeutic strategies found no significant association between this SNP and survival or time to cancer recurrence [70,71]. Finally, no other studies evaluating the influence of the *ERCC1* rs10412761 SNP on the effectiveness of chemotherapy was found.

The differences observed in the results obtained for certain SNPs with respect to their impact on toxicity or effectiveness of capecitabine-based therapy among the studies included in this review may be due to several factors, including (a) high methodological diversity among studies, such as design, population ethnicity, sample size, clinical data collection, response or toxicity assessment methods, and capecitabine therapeutic regimens evaluated, as well as (b) differences in the statistical methodology applied to control for confounding factors and whether or not adjustments for multiple comparisons were applied.

This review has some limitations that need to be mentioned: (a) It only included studies that analyzed patients with CRC under capecitabine-based treatment, and this substantially reduced the possible number of results and impedes their generalizability to other FPs and neoplasms. (b) Moreover, it only examined the influence of SNPs on the effectiveness and safety of capecitabine-based therapy, excluding the possible effect of other genetic variants that have been associated with effects on capecitabine therapy outcomes (tandem repeats, copy number variations, insertions, deletions, etc.). (c) Due to the high variety of SNPs reviewed, it was not possible to perform a meta-analysis to observe variations in the level of association of genotypes with the outcomes of capecitabine-based therapy. (d) A large proportion of the SNPs examined for each gene were reported by only one study, revealing the need for further evidence to corroborate these findings. (e) The included studies were in the moderate to low range of methodological quality according to the STREGA statement criteria, thus interpretations of the findings of this review must be treated with caution.

## 5. Conclusions

In conclusion, the results obtained in this systematic review suggest that according to the recent literature, as well as the four SNPs of current clinical relevance in *DPYD*, there are other SNPs in genes related to the PD (*TYMS*, *ENOSF1*, *MTHFR*, *ERCC1*, and *ERCC2*) and PK (*CDA*, *DPYD*, *UMPS*, and *SLC22A7*) of capecitabine that could come to be considered in the future as predictive biomarkers of the outcomes of capecitabine-based therapy in patients with CRC. Specifically, current evidence suggests that SNPs *TYMS/ENOSF1* rs699517, *ENOSF1* rs2612091, and *CDA* rs2072671 appear to have a nearer future as biomarkers in clinical practice, although they still require further prospective validation. The remaining SNPs in the aforementioned genes require additional studies to elucidate their influence on capecitabine toxicity and effectiveness in CRC patients.

## Figures and Tables

**Table 1:** Characteristics of the studies included.

First Author (Year) Country	Study Design	Clinical Data Collection	Ethnicity	N	Median Age (Range)	Women (%)	CRC Stage	Treatment Regimens	Treatment Setting	Genes of Interest Investigated	Object of Study	Ref.
Van Huis-Tanja LH (2013) Netherlands	Cohort of an RCT	Prospective	European ancestry	126	61 (27–78)	49 (39)	IV	CAPE	MX	*MTHFR*	Effectiveness/Toxicity	[20]
Rosmarin D (2015) UK	Cohort of an RCT	Prospective	European ancestry	940	65 (22–85)	453 (43)	II–III	CAPE, CAPE-B	ADJ	*ABCC3, ABCC4, ABCC5, ABCG2, ABCB1, CDA, CES1, CES2, DPYD, DPYS, MTHFR, PPAT, RRM1, RRM2, SLC22A7, SLC29A1, TK1, TYMP, TYMS, UCK1, UCK2, UMPS, UPB1, UPP1, UPP2*	Toxicity	[21]
García-González X (2015) Spain	Cohort	Ambispective	NR	239	67 (30–88)	110 (46)	I–IV	CAPE, CAPOX, CAPIRI, CAPE-AB	ADJ, MX	*ABCB1, CDA, ENOSF1, MTHFR, TYMS*	Toxicity	[22]
Falvella FS (2015) Italy	Cohort of 2 RCTs	Prospective	European ancestry	64	57 (34–73)	25 (39)	IV	CAPOXIRI-B, CAPOXIRI-CETU	MX	*DPYD*	Toxicity	[23]
Sebio A (2015) Spain	Cohort	NR	European ancestry	84	68 (80–42)	29 (35)	II–III	CAPE-RT	NEOADJ	*ERCC1, ERCC2, TYMS, XRCC1*	Effectiveness	[24]
Pellicer M (2017a) Spain	Cross- sectional	Ambispective	European ancestry	301	65 (30–88)	145 (48)	I–IV	CAPE, CAPOX, CAPIRI, CAPE-AB	NEOADJ, ADJ, MX	*CDA, CES1, DPYD, ENOSF1, SLC22A7, TYMP, TYMS, TYMS/ENOSF1, ENOSF1, UMPS*	Toxicity	[25]
Pellicer M (2017b) Spain	Cohort	NR	European ancestry	319	65 (30–88)	151 (47)	I–IV	CAPE, CAPOX, CAPIRI, CAPE-AB	NEOADJ, ADJ, MX	*ABCC4, DPYD, MTHFR*	Toxicity	[26]
Matevska-Geshkovska, N (2018) Macedonia	OLCT	Prospective	NR	126	60 (36–81)	50 (45)	II–III	CAPE	ADJ	*MTHFR, TYMS*	Effectiveness	[27]
Varma A (2019) India	Cohort	Prospective	Asian: Tamilian (76.5%), Andhra (14.4%), N. Indians (9.6%)	145	50 (NR)	55 (38)	II–IV	CAPOX	NEOADJ, ADJ, P	*DPYD*	Toxicity	[28]
Boige V (2019) France	Cohort of an RCT	Prospective	European ancestry	316	61 (35–79)	104 (33)	II–III	CAPOX-RT, CAPE-RT	NEOADJ	*ERCC1, ERCC2, MTHFR, TYMS, XRCC1, XRCC3*	Effectiveness/Toxicity	[29]
Varma A (2020) India	Cohort	Prospective	Asian: Tamilian (76.5%), Andhra (14.4%), Kerala (9.6%)	145	50 (NR)	55 (38)	II–IV	CAPOX	ADJ	*ABCB1, ERCC1, ERCC2*	Effectiveness	[30]
Puerta-García E (2020) Spain	Cohort	Retrospective	European ancestry	84	68 (60–72)	30 (35)	I–IV	CAPE	ADJ	*ABCB1, DPYD, MTHFR, ERCC1, XRCC1*	Toxicity	[31]
Dong SQ (2021) China	Case-control	Retrospective	Asian	288	59 (27–83)	108 (38)	I–IV	CAPE, CAPOX, CAPIRI, CAPE-AB	NEOADJ, ADJ, MX	*TYMS*	Toxicity	[32]

AB: antibodies, ADJ: adjuvant, B: bevacizumab, CAPE: capecitabine, CAPIRI: capecitabine + irinotecan, CAPOX: capecitabine + oxaliplatin, CAPOXIRI: capecitabine + oxaliplatin + irinotecan, CETU: cetuximab, MX: metastatic, NEOADJ: neoadjuvant, NR: not reported, OLCT: open-label clinical trial, P: palliative, RCT: randomized controlled trial, RT: radiotherapy.

**Table 2:** Single-nucleotide polymorphisms associated with capecitabine-induced toxicity in colorectal cancer patients.

Variant rs Number	SNP Position	Variant Type/Consequence	Associated Genotype/Allele vs. Reference	Toxicity			Ref.
				Grade (Type)	*p* Value ^a^	OR (95% CI)	
Gene *CDA*							
rs2072671	c.79A>C	missense	AA vs. AC-CC	3–4 (diarrhea) 2–4 (HFS) 3–4 (HFS) 3–4 (hematological) 3–4 (asthenia) 3–4 (overall)	0.157 0.163 0.066 0.531 0.566 0.029	1.83 (0.79–4.24) 1.56 (0.83–2.94) 2.89 (0.93–8.98) 1.38 (0.50–3.80) 1.40 (0.44–4.49) 1.84 (1.06–3.18)	[22]
			AC-CC vs. AA	3–4 (HFS) 3–4 (overall)	0.008 0.007	0.27 (0.10–0.71) 0.50 (0.30–0.83)	[25]
rs1048977	c.435C>T	synonymous	CT-TT vs. CC	3–4 (hyperbilirubinemia)	0.044	8.62 (1.05–70.24)	[25]
*DPYD*							
rs3918290	c.IVS14+1G>A	splice donor	AG vs. GG	3–4 (overall)	0.179 ^b^	3.02 (0.50–18.15)	[21]
rs55886062	1679T>G	missense	GT vs. TT	3–4 (overall)	0.697 ^b^	4.02 (0.36–44.47)	[21]
rs67376798	c.2846A>T	missense	AT vs. AA	3–4 (overall)	0.001 ^b^	8.17 (1.73–38.70)	[21]
			AT vs. AA	3–4 (overall) 1–4 (GI) 1–4 (cardiovascular) 1–4 (asthenia) 1–4 (cutaneous) 1–4 (respiratory)	0.287 ^c^ 1.000 ^c^ 1.000 ^c^ 0.250 ^c^ 0.412 ^c^ 0.125 ^d^	U U 0.00 (0.00–NaN) U U U	[31]
rs56038477	c.1236G>A	synonymous	AG vs. GG	3–4 (overall)	0.008 ^b^	2.73 (1.38–5.41)	[21]
rs1801160	c.2194G>A	missense	AG-AA vs. GG	3–4 (overall)	0.827 ^b^	1.16 (0.69–1.96)	[21]
			AG-AA vs. GG	2–4 (overall)	0.029	2.11 (1.08–4.13)	[26]
			AG-AA vs. GG	1–4 (anemia) 1–4 (thrombocytopenia) 1–4 (neutropenia) 1–4 (vomiting) 1–4 (diarrhea) 1–4 (HFS) 1–4 (PN)	0.800 ^e^ 0.600 ^e^ 0.200 ^e^ 0.900 ^e^ 0.100 ^e^ 0.300 ^e^ 0.700 ^e^	1.90 (0.40–2.60) 1.20 (0.50–3.10) 1.70 (0.60–1.70) 1.00 (0.40–2.00) 1.80 (0.70–2.00) 0.60 (0.20–1.00) 1.10 (0.40–2.00)	[28]
rs1801265	c.85T>C	missense	CT-CC vs. TT	3–4 (overall)	0.078 ^b^	0.76 (0.58–1.01)	[21]
			CT-CC vs. TT	1–4 (anemia) 1–4 (thrombocytopenia) 1–4 (neutropenia) 1–4 (vomiting) 1–4 (diarrhea) 1–4 (HFS) 1–4 (PN)	0.800 ^e^ 0.010 ^e^ 0.500 ^e^ 0.060 ^e^ 0.040 ^e^ 0.020 ^e^ 0.900 ^e^	0.90 (0.40–1.80) 2.40 (1.18–5.10) 1.24 (0.50–2.90) 1.00 (0.90–4.00) 2.70 (1.80–4.00) 2.30 (1.80–4.00) 0.90 (0.40–2.00)	[28]
rs1801158	c.1601G>A	missense	AG vs. GG	3–4 (overall)	0.368 ^b^	1.38 (0.73–2.59)	[21]
rs1801159	c.1627A>G	missense	AG-GG vs. AA	3–4 (overall)	0.560 ^b^	1.03 (0.77–1.36)	[21]
rs2297595	c.496A>G	missense	AG-GG vs. AA	3–4 (overall)	0.415 ^b^	0.92 (0.64–1.31)	[21]
			AG vs. AA	3–4 (overall)	0.022	5.94 (1.29–27.22)	[23]
rs12022243	c.1906-14763G>A	intron	A vs. G	3–4 (overall) 3–4 (HFS) 3–4 (diarrhea)	2.55 × 10^−5 b^ 0.009 ^b^ 9.86 × 10^−6 b^	1.69 (1.45–1.94) 1.43 (1.16–1.70) 1.79 (1.54–2.05)	[21]
rs7548189	C.1906-19696G>T	intron	T vs. G	3–4 (overall) 3–4 (HFS) 3–4 (diarrhea) 2–4 (diarrhea)	3.79 × 10^−5 b^ 0.011 ^b^ 0.001 ^b^ 1.72 × 10^−5 b^	1.67 (1.43–1.91) 1.42 (1.15–1.69) 1.21 (0.84–1.58) 1.76 (1.50–2.02)	[21]
rs45589337	c.775A>G	missense	AG vs. AA	3–4 (overall)	0.723 ^b^	0.80 (0.25–2.56)	[21]
rs76387818	g.97539400G>A	–	A vs. G	3–4 (overall) 3–4 (HFS) 3–4 (diarrhea)	2.11 × 10^−6 b^ 1.75 × 10^−8 b^ 0.071 ^b^	4.05 (3.47–4.62) 6.44 (5.79–7.09) 0.44 (0.00–1.33)	[21]
rs12132152	g.97523004G>A	–	A vs. G	3–4 (overall) 3–4 (HFS) 3–4 (diarrhea)	4.31 × 10^−6 b^ 3.29 × 10^−8 b^ 0.065 ^b^	3.83 (3.26–4.40) 6.12 (5.48–6.76) 0.44 (0.00–1.32)	[21]
rs17376848	1896T>C	synonymous	CT vs. TT	3–4 (overall)	0.027	14.53 (1.36–155.20)	[23]
			CT vs. TT	3–4 (overall) 1–4 (GI) 1–4 (cardiovascular) 1–4 (asthenia) 1–4 (skin) 1–4 (respiratory)	0.494^c^ 1.000 ^c^ 1.000 ^c^ 1.000 ^c^ 1.000 ^c^ 1.000 ^c^	2.51 (0.03–202.96) U 0.00 (0.00–NaN) 0.00 (0.00–NaN) 1.43 (0.02–115.15) 0.00 (0.00–NaN)	[31]
rs12119882	c.680+2545T>C	intron	CT-CC vs. TT	3–4 (hyperbilirubinemia)	0.031	4.86 (1.16–20.38)	[25]
*UMPS*							
rs4678145	c.156+607G>C	intron	CG-CC vs. GG	3–4 (asthenia)	0.006	4.54 (1.55–13.24)	[25]
rs2279199	c.-67T>C	2KB upstream	CT-CC vs. TT	3–4 (nausea and vomiting)	0.036	0.21 (0.04–0.90)	[25]
*SLC22A7*							
rs2270860	1269C>T	synonymous	TT vs. CT-CC	3–4 (skin)	0.016	17.08 (1.71–170.26)	[25]
rs4149178	1592+206A>G	intron	AG-GG vs. AA	3–4 (diarrhea)	0.034	0.34 (0.12–0.92)	[25]
*ABCB1*							
rs1128503	c.1236T>C	synonymous	CC vs. CT-TT	3–4 (overall) 1–4 (GI) 1–4 (cardiovascular) 1–4 (asthenia) 1–4 (skin) 1–4 (respiratory)	0.044 ^d^ 0.643 ^d^ 0.562 ^d^ 0.372 ^d^ 0.402 ^d^ 1.000 ^c^	0.22 (0.02–1.11) 0.77 (0.24–2.72) 1.63 (0.03–33.00) 0.49 (0.08–2.04) 0.66 (0.22–1.92) 0.88 (0.13–4.30)	[31]
*TYMS*							
rs2853741	c.-391T>C	2KB upstream	CC vs. CT-TT	3–4 (diarrhea)	0.008	0.31 (0.13–0.74)	[25]
rs3786362	c.381A>G	synonymous	AA vs. AG vs. GG	2–3 (HFS)	1.89 × 10^−3^	0.38 (0.21–0.70)	[32]
*TYMS/ENOSF1*							
rs699517	c.*19C>T	3′UTR/noncoding transcript	TT vs. CT-CC	3–4 (nausea and vomiting) 3–4 (anorexia)	0.014 0.006	7.93 (1.51–41.63) 128.82 (4.16–3988.96)	[25]
			CT-TT vs. CC	3–4 (asthenia)	0.021	0.24 (0.07–0.81)	[25]
			CC vs. CT vs. TT	2–3 (HFS)	4.62 × 10^−4^	2.12 (1.39–3.24)	[32]
rs2790	c.*89A>G	3′UTR/intron	AA vs. AG vs. GG	2–3 (HFS)	8.80 × 10^−3^	0.58 (0.39–0.87)	[32]
*ENOSF1*							
rs2612091	c.496-227G>A	intron	G vs. A	3–4 (overall) 3–4 (HFS) 3–4 (diarrhea)	5.28 × 10^−6 b^ 2.94 × 10^−6 b^ 0.290 ^b^	1.59 (1.39–1.79) 1.57 (–) 1.18 (0.55–1.15)	[21]
			GG vs. GA-AA	3–4 (diarrhea) 2–4 (HFS) 3–4 (HFS) 3–4 (hematological) 3–4 (asthenia) 3–4 (overall)	0.431 0.027 0.114 0.541 0.063 0.789	0.60 (0.17–2.12) 2.28 (1.10–4.76) 2.53 (0.80–8.02) 0.62 (0.14–2.84) 3.15 (0.94–10.57) 0.91 (0.45–1.82)	[22]
rs2741171	c.63+5783A>G	intron	A vs. G	3–4 (overall) 3–4 (HFS) 3–4 (diarrhea)	6.64 × 10^−6 b^ 1.64 × 10^−6 b^ 0.920 ^b^	1.60 (1.39–1.80) 1.74 (1.51–1.97) 1.01 (0.70–1.32)	[21]
*MTHFR*							
rs1801131	c.1286A>C	missense	CC vs. AC-AA	3–4 (overall) 3–4 (diarrhea) 3–4 (HFS)	0.355 ^f^ 0.041 ^f^ 0.406 ^f^	1.85 (0.55–6.11) 6.00 (1.28–28.09) 1.90 (0.47–7.75)	[20]
			CC vs. AC-AA	3–4 (overall) 1–4 (GI) 1–4 (cardiovascular) 1–4 (asthenia) 1–4 (skin) 1–4 (respiratory)	0.529 ^d^ 1.000 ^c^ 1.000 ^c^ 0.741 ^c^ 0.464 ^d^ 0.682 ^c^	1.47 (0.34–5.73) 1.24 (0.31–6.07) – 1.24 (0.25–5.12) 1.52 (0.40–5.79) 0.49 (0.01–4.13)	[31]
rs1801133	c.665C>T	missense	TT vs. CT-CC	3–4 (overall) 3–4 (diarrhea) 3–4 (HFS)	0.770 ^f^ 0.596 ^f^ 0.237 ^f^	1.35 (0.44–4.17) 0.00 (0.00–NaN) 2.40 (0.67–8.59)	[20]
			TT vs. CT-CC	3–4 (overall) 1–4 (GI) 1–4 (cardiovascular) 1–4 (asthenia) 1–4 (skin) 1–4 (respiratory)	0.403 ^c^ 0.676 ^c^ 1.000 ^c^ 0.009 ^c^ 0.693 ^c^ 0.209 ^c^	1.95 (0.26–12.79) 0.61 (0.09–4.56) – 9.30 (1.36–106.8) 0.55 (0.05–3.61) 3.18 (0.26–23.9)	[31]

GI: gastrointestinal; HFS: hand–foot syndrome; NaN: not a number; OR: odds ratio; PN: peripheral neuropathy; Ref: reference category; SNP: single-nucleotide polymorphism; U: undefined. ^a^ *p* value for multivariate logistic regression; ^b^ *p* value for Bonferroni correction threshold of 3.43 × 10^–5^; ^c^ *p* value for Fisher exact test; ^d^ *p* value for Bonferroni correction threshold of 0.01; ^e^ *p* value for χ2 test; ^f^ *p* value for χ2 test with significance level corrected to 0.01.

**Table 3:** Single-nucleotide polymorphisms associated with capecitabine effectiveness in colorectal cancer patients.

Variant rs Number	SNP Position	Variant Type/Consequence	Associated Genotype/Allele vs. Reference	Effectiveness Outcomes						Ref.
				PFS		Response		OS		
				*p* Value ^a^	HR (95% CI)	*p* Value ^c^	OR (95% CI)	*p* Value	HR (95% CI)	
Gene										
*ABCB1*										
rs1128503	c.1236T>C	synonymous	CT-CC vs. TT	-	–	0.040 ^d^	3.70 (0.70–19.00)	–	–	[30]
rs1045642	c.3435T>C	synonymous	CT-CC vs. TT	-	–	0.050 ^d^	3.10 (0.80–13.00)	–	–	[30]
*ERCC1*										
rs11615	c.354T>C	synonymous	CC vs. CT-TT	-	–	0.023	NE	–	–	[24]
			CT-CC vs. TT	-	–	0.300 ^d^	0.50 (0.10–2.00)	–	–	[30]
rs10412761	g.45908461A>G	–	AG-GG vs. AA	-	–	0.042	0.57 (0.34–0.98)	0.160	1.47 (0.85–2.56)	[29]
*ERCC2*										
rs13181	c.2251A>C	stop gained	AC-CC vs. AA	-	–	–	–	0.235	0.73 (0.43–1.22)	[29]
			AC-CC vs. AA	-	–	0.500 ^d^	0.80 (0.10–4.00)	–	–	[30]
rs1799787	c.1832-70C>T	intron	CT-TT vs. CC	-	–	0.027	0.55 (0.33–0.93)	0.276	0.75 (0.45–1.25)	[29]
*MTHFR*										
rs1801131	1286A>C	missense	CC vs. AC-AA	0.904 ^b^	–	0.691	–	0.758	–	[20]
rs1801133	665C>T	missense	TT vs. CT-TT	0.807 ^b^	–	0.127	–	0.270	–	[20]
			TT vs. CT-TT	0.225	0.29 (0.04–2.13)	–	–	–	–	[27]
rs7553194	c.-578C>T	noncoding transcript	CT-TT vs. CC	-	–	–	–	0.108	0.49 (0.20–1.26)	[29]

HR: hazard ratio; NE: not estimable; OR: odds ratio; OS: overall survival; Ref: reference category; PFS: progression-free survival; SNP: single-nucleotide polymorphism. ^a^ *p* value for multivariate Cox proportional hazards regression model; ^b^ *p* value for Mann–Whitney U test; ^c^ *p* value for multivariate logistic regression; ^d^ significance level defined as <0.01.

## Data Availability

All data can be found in the text.

## Supplementary Materials

The following supporting information can be downloaded at https://www.mdpi.com/article/10.3390/cancers15061821/s1: Table S1. Complete search strategy; Table S2. Reporting quality of the studies included.

## Abbreviations

5,10-MTHF: 5,10-methylenetetrahydrofolate; 5-FU: 5-fluorouracil; 5′-dFCR: 5′-deoxy-5-fluorocytidine; 5′-dFUR: 5′-deoxy-5-fluorouridine; ABCB1: ATP-binding cassette subfamily B member 1; ABCC3: ATP-binding cassette subfamily C member 3; ABCC4: ATP-binding cassette subfamily C member 4; ABCC5: ATP-binding cassette subfamily C member 5; ABCG2: ATP-binding cassette subfamily G member 2; ATase: amidophosphoribosyltransferase; CAPOX: capecitabine + oxaliplatin; CAPOXIRI: capecitabine + oxaliplatin + irinotecan; CDA: cytidine deaminase; CES1: carboxylesterase 1; CES1P1: carboxylesterase 1 pseudogene 1; CES2: carboxylesterase 2; CRC: colorectal cancer; CTCAE: Common Terminology Criteria for Adverse Events; DHFR: dihydrofolate reductase; DHFU: dihydrofluorouracil; DPD: dihydropyrimidine dehydrogenase (enzyme); DPYD: dihydropyrimidine dehydrogenase (gene); DPYS: dihydropyrimidinase; ENOSF1: enolase superfamily member 1; ENT1: equilibrative nucleoside transporter 1; ERCC1: ERCC excision repair 1; ERCC2: ERCC excision repair 2; FBAL: fluoro-beta-alanine; FdUMP: fluorodeoxyuridine monophosphate; FdUTP: fluorodeoxyuridine triphosphate; FP: fluoropyrimidine; FPGS: folylpolyglutamate synthase; FUDP: fluorouridine diphosphate; FUTP: fluorouridine triphosphate; GGH: gamma-glutamyl hydrolase; HFS: hand-foot syndrome; HWE: Hardy–Weinberg equilibrium; IARC: International Agency for Research on Cancer; LD: linkage disequilibrium; MDR1: multidrug resistance 1; MTHFD1, methylenetetrahydrofolate dehydrogenase 1; MTHFR: methylenetetrahydrofolate reductase; NSCLC: non-small-cell lung cancer; OAT2: organic anion transporter 2; ORR: overall response rate; OS: overall survival; PD: pharmacodynamics; PFS: progression-free survival; P-gp: P-glycoprotein; PGx: pharmacogenetics; PK: pharmacokinetics; PPAT: phosphoribosyl pyrophosphate amidotransferase; PRISMA: Preferred Reporting Items for Systematic Reviews and Meta-Analyses; RNR: ribonucleotide reductase; RRM1: ribonucleotide reductase catalytic subunit M1; RRM2: ribonucleotide reductase regulatory subunit M2; RT: radiotherapy; SHMT1: serine hydroxymethyltransferase 1; SLC22A7: solute carrier family 22 member 7; SLC29A1: solute carrier family 29 member 1; SMUG1: single-strand-selective monofunctional uracil-DNA glycosylase 1; SNP: single-nucleotide polymorphism; STREGA: Strengthening the Reporting of Genetic Association studies; TDG: thymine DNA glycosylase; TK: thymidine kinase (enzyme); TK1: thymidine kinase 1 (gene); TP: thymidine phosphorylase (enzyme); TP53: tumor protein p53; TS: thymidylate synthase (enzyme); TYMP: thymidine phosphorylase (gene); TYMS: thymidylate synthetase (gene); UCK: uridine–cytidine kinase; UCK1: uridine–cytidine kinase 1; UCK2: uridine–cytidine kinase 2; UMPS: uridine monophosphate synthetase; UPB1: beta-ureidopropionase 1; UPP: uridine phosphorylase; UPP1: uridine phosphorylase 1; UPP2: uridine phosphorylase 2; XRCC1: X-ray repair cross complementing 1; XRCC3: X-ray repair cross complementing 3.
