# Supplementary material for: Influence of Single-Nucleotide Polymorphisms on Clinical Outcomes of Capecitabine-Based Chemotherapy in Colorectal Cancer Patients: A Systematic Review
Source: Cancers (Basel). 2023 Mar 17;15(6):1821. doi: 10.3390/cancers15061821 (PMC10046456; doi:10.3390/cancers15061821)
Supplement: Supplementary file 1 [file cancers-15-01821-s001.zip › cancers-2233031-supplementary.pdf]

Supplementary material

Table S1. Complete search strategy.

Search Strategy
((CES1 OR CES1A1 OR Carboxylesterase-1) OR (CES1P1 OR CES1A2 OR Carboxylesterase-1-Pseudogene-1) OR (CES2 OR CES2A1 OR Carboxylesterase-2) OR (CDA OR CDD OR Cytidine-Deaminase) OR (DPYD OR DPD OR DHP OR Dihydropyrimidine-Dehydrogenase) OR (DPYS OR DHP OR Dihydropyrimidinase) OR (PPAT OR GPAT OR Phosphoribosyl-Pyrophosphate-Amidotransferase) OR (RRM2 OR RR2 OR Ribonucleotide-Reductase-Regulatory-Subunit-M2) OR (RRM1 OR RR1 OR Ribonucleotide-Reductase-Catalytic-Subunit-M1) OR (TK1 OR Thymidine-Kinase-1) OR (TYMP OR TP OR Thymidine-Phosphorylase) OR (UCK1 OR URK1 OR Uridine-Cytidine-Kinase-1) OR (UCK2 OR UMPK OR Uridine-Cytidine-Kinase-2) OR (UMPS OR OPRT OR Uridine-Monophosphate-Synthetase) OR (UPP1 OR Uridine-Phosphorylase-1) OR (UPP2 OR Uridine-Phosphorylase-2) OR (UPB1 OR Beta-Ureidopropionase-1) OR (ABCC3 OR ATP-Binding-Cassette-Subfamily-C-Member-3) OR (ABCC4 OR ATP-Binding-Cassette-Subfamily-C-Member-4) OR (ABCC5 OR ATP-Binding-Cassette-Subfamily-C-Member-5) OR (ABCG2 OR BCRP OR ATP-Binding-Cassette-Subfamily-G-Member-2) OR (SLC22A7 OR OAT2 OR Solute-Carrier-Family-22-Member-7) OR (SLC29A1 OR Solute-Carrier-Family-29-Member-1) OR (ABCB1 OR PG-P OR MDR1 OR ATP-Binding-Cassette-Subfamily-B-Member-1) OR (TYMS OR TS OR Thymidylate-Synthetase) OR (ENOSF1 OR Enolase-Superfamily-Member-1) OR (MTHFR OR Methylenetetrahydrofolate-Reductase) OR (DHFR OR Dihydrofolate-Reductase) OR (MTHFD1 OR Methylenetetrahydrofolate-Dehydrogenase) OR (SHMT1 OR Serine-Hydroxymethyltransferase-1) OR (GGH OR Gamma-Glutamyl-Hydrolase) OR (FPGS OR Folylpolyglutamate-Synthase) OR (ERCC2 OR XPD OR ERCC-Excision-Repair-2) OR (ERCC1 OR ERCC-Excision-Repair-1) OR (SMUG1 OR Single-Strand-Selective-Monofunctional-Uracil-DNA-Glycosylase-1) OR (TDG OR Thymine-DNA-Glycosylase) OR (XRCC3 OR X-Ray-Repair-Cross-Complementing-3) OR (XRCC1 OR RCC OR X-Ray-Repair-Cross-Complementing-1) OR (TP53 OR P53 OR Tumor-Protein-P53) AND capecitabine AND (colon OR colonic OR colorectal OR rectal) AND (neoplasm OR cancer OR carcinoma OR malignant OR malignancy))

Table S2. Reporting quality of the included studies.

Study	Item 1	Item 2	Item 3	Item 4	Item 5	Item 6	Item 7	Item 8	Item 9	Score (%)
Van Huis-Tanja L, H (2013)	Y	Y	N	N	Y	Y	N	Y	Y	6.00 (66.67)
Rosmarin D (2015)	I	Y	N	N	Y	Y	Y	Y	Y	6.50 (72.22)
García-González X (2015)	Y	N	N	N	Y	N	Y	Y	Y	5.00 (55.56)
Falvella FS (2015)	I	I	N	N	Y	N	N	Y	Y	4.00 (44.40)
Sebio A (2015)	I	N	N	N	I	Y	Y	Y	Y	5.00 (55.56)
Pellicer M (2017a)	Y	Y	Y	N	Y	N	N	N	Y	5.00 (55.60)
Pellicer M (2017b)	Y	N	N	N	Y	N	N	N	Y	3.00 (33.33)
Matevska-Geshkovska, N (2018)	I	N	N	Y	N	Y	N	N	Y	3.50 (38.89)
Varma A (2019)	Y	N	N	N	Y	N	N	Y	Y	4.00 (44.44)
Boige V (2019)	I	N	N	N	I	Y	Y	Y	Y	5.00 (55.64)
Varma A (2020)	Y	N	N	N	Y	N	N	Y	Y	4.00 (44.44)
Puerta-García E (2020)	I	N	N	N	Y	Y	N	Y	Y	4.50 (50.00)
Dong SQ (2020)	I	N	N	N	I	Y	Y	N	Y	4.00 (44.44)

I, incomplete. N, no. Y, yes. Item description: Item 1. Describe laboratory methods, including source and storage of DNA, genotyping methods and platforms; Item 2: Describe error rate and call rate; Item 3: State the laboratory/center at which genotyping was performed; Item 4: Mentions whether the genotypes were assigned in one single batch or a few smaller batches; Item 5: Report number of individuals in whom genotyping was attempted to and how many of these samples were successfully genotyped; Item 6: Describe methods used to assess/address population stratification; Item 7: Describe any methods used for inferring genotypes or haplotypes; Item 8: Stated whether Hardy–Weinberg equilibrium was considered; Item 9: State if the study is the first report to report such genetic association, a replication or both.
